# Differential contribution of electrically evoked dorsal root reflexes to peripheral vasodilatation and plasma extravasation

**DOI:** 10.1186/1742-2094-8-20

**Published:** 2011-02-28

**Authors:** Oleg V Lobanov, Yuan B Peng

**Affiliations:** 1 Department of Psychology, University of Texas at Arlington, Arlington, TX 76019, USA

## Abstract

**Background:**

Dorsal root reflexes (DRRs) are antidromic activities traveling along the primary afferent fibers, which can be generated by peripheral stimulation or central stimulation. DRRs are thought to be involved in the generation of neurogenic inflammation, as indicated by plasma extravasation and vasodilatation. The hypothesis of this study was that electrical stimulation of the central stump of a cut dorsal root would lead to generation of DRRs, resulting in plasma extravasation and vasodilatation.

**Methods:**

Sprague-Dawley rats were prepared to expose spinal cord and L4-L6 dorsal roots under pentobarbital general anesthesia. Electrical stimulation of either intact, proximal or distal, cut dorsal roots was applied while plasma extravasation or blood perfusion of the hindpaw was recorded.

**Results:**

While stimulation of the peripheral stump of a dorsal root elicited plasma extravasation, electrical stimulation of the central stump of a cut dorsal root generated significant DRRs, but failed to induce plasma extravasation. However, stimulation of the central stump induced a significant increase in blood perfusion.

**Conclusions:**

It is suggested that DRRs are involved in vasodilatation but not plasma extravasation in neurogenic inflammation in normal animals.

## Background

Somatosensory information is generally considered to originate in the peripheral terminals of primary afferent neurons, and is then transmitted to the spinal cord or brain. However, activity in primary afferent neurons can be generated within the spinal cord, and the impulses can travel antidromically toward the periphery. These phenomena are called dorsal root reflexes (DRR). Dorsal root reflexes were first discovered by Gotch and Horsley [1], and were studied extensively in later works [2-9].

DRRs are thought to contribute to neurogenic inflammation. The main components of neurogenic inflammation include, but are not limited to, arteriolar vasodilatation and plasma extravasation. Neurogenic inflammation is triggered by substances released from sensory nerve terminals, including substance P (SP) and calcitonin gene-related peptide (CGRP). SP, as well as other tachykinins such as neurokinin A (NKA) and neurokinin B (NKB), cause plasma extravasation by a specific action on NK_1_, NK_2_, and NK_3 _receptors [10] to increase vascular permeability [11-13]. SP and NKA play a major role in the periphery, whereas NKB is mainly found in the CNS [14]. CGRP is active in dilating cutaneous arterioles [15] via the CGRP_1 _receptor [11,13]. Both tachykinins and CGRP are found in the peripheral endings of sensory nerves [16-19] and released from both C and Aδ fibers [11].

Neurogenic inflammation has been shown to be caused by antidromic electrical stimulation of afferent nerves [20-26], by intradermal injection of capsaicin [27], and in acute [28-31] or chronic [32] arthritis experiments. Enhanced afferent discharges cause the central terminals of primary afferent fibers to release excitatory amino acids, which then activate non-NMDA and NMDA receptors on GABAergic interneurons, leading to the release of GABA on primary afferent central terminals [33-36]. GABA produces excessive primary afferent depolarization (PAD) through GABA_A _receptors located on presynaptic terminals of primary afferents [37,38]. When PAD exceeds the threshold, DRRs are generated [39,40], which are conducted antidromically in both myelinated and unmyelinated fibers toward the periphery [41,42], and can be blocked by the spinal GABA_A _antagonist, bicuculline [43]. This antidromic activity could result in the release of inflammatory mediators (e.g., SP), as was shown in the knee joint [23,44].

DRRs can be induced by electrical stimulation of peripheral nerves, both ipsilaterally and contralaterally [45], as well as by supraspinal stimulation of the periaqueductal grey [46]. In the present study we tried to mimic the incoming nociceptive input to the spinal cord by electrical stimulation of the central stump of the dorsal root, and test whether the electrically evoked DRRs can contribute to the development of neurogenic inflammation - vasodilatation and plasma extravasation. Preliminary results have been reported [47].

## Methods

### Animal preparation

A total of 19 adult male Sprague-Dawley rats weighing 300-400 g were used for this study, 8 for vasodilatation, and 11 for plasma extravasation. All procedures used in this study were approved by the Animal IACUC and followed the guidelines for the treatment of animals of the International Association for the Study of Pain [48].

Animals were initially anesthetized with sodium pentobarbital (50 mg/kg, i.p.). A catheter was placed into the jugular vein for continuous administration of anesthetic (sodium pentobarbital, 5-8 mg·kg^-1^·h^-1 ^in a saline solution) and for Evans Blue injection in plasma extravasation experiments. The level of anesthesia was monitored by the stability of the level of end-tidal CO_2 _at around 30 mmHg and by the absence of flexion reflex. Tracheotomy was performed for artificial ventilation. The animal's body temperature was maintained at 37°C by a feedback controlled electric heating blanket. A 4-cm-long laminectomy was performed over the lumbosacral enlargement to expose the spinal cord and L4-L6 dorsal roots. The rat was held in a stereotaxic frame to prevent movement during recording. The skin over the laminectomy formed a pool and was filled with light mineral oil.

### DRR recordings

**Figure 1:**
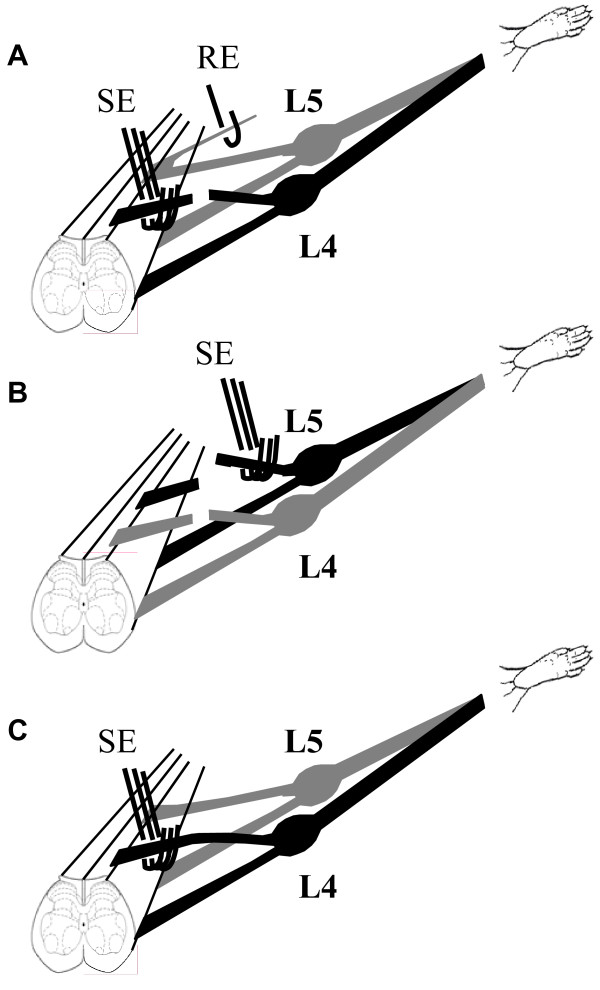
**Diagram of the experimental setup**. A. The left L4 dorsal root is cut. The central stump is placed over a stimulating electrode (SE) while placing a strand from the left L5 dorsal root on a recording electrode (RE). B. A second cut is made at the left L5 dorsal root. The peripheral stump of the left L5 dorsal root is placed over a stimulating electrode. Seven minutes after Evans Blue injection (i.v.), electrical stimulation (20 V, 5 Hz, 0.5 ms for 5 min) is delivered, while a series of images is taken. C. A setup for stimulating an intact dorsal root.

A silver wire hook electrode was used to record extracellular single-unit discharges in filaments of the L4 through L6 dorsal roots. A small strand of the dorsal root was teased centrally from the main trunk and was further separated into a fine filament containing one or a few active fibers. This filament was then wrapped around the recording electrode (Figure 1A).

Data recording and analysis was performed by using a CED 1401Plus data acquisition system and SPIKE2 software (Cambridge Electronic Design Ltd, UK).

### Electrical stimulation

Electrical stimulation of the central stump was performed in 16 animals, 8 for plasma extravasation and 8 for laser Doppler measurement. A tripolar electrode was used for electrical stimulation in order to minimize stimulus artifact and to avoid current spread. The cathode was in the middle of the array, and two anodes, one on each side of the cathode, were separated from the cathode by 1 mm [46]. The L4 or L5 dorsal roots were cut and the central stump was placed on the electrode for electrical stimulation (Figure 1A), while a teased strand from a nearby intact dorsal root was used for recording DRRs. Then stimulation was applied to this central stump of the cut dorsal root for 5 minutes at 20 V, 5 Hz, 0.5 ms pulse duration.

At the end of stimulation of the central stump (L4 or L5) for plasma extravasation, the dorsal root that was used for DRR recording (L5 or L6) was cut and the peripheral stump was stimulated at the same parameters (20 V, 5 Hz, 0.5 ms; Figure 1B).

In 3 animals, intact L4 or L5 dorsal root was stimulated at the same parameters (20 V, 5 Hz, 0.5 ms; Figure 1C)

### Plasma extravasation measurement

Plasma extravasation measurements were performed in 11 animals, 8 for central and peripheral stump stimulation (Figure 1A) and 3 for intact dorsal root stimulation (Figure 1C). Evans Blue was injected intravenously (50 mg/kg, using the catheter in the jugular vein) for detection of the sites of plasma extravasation 7 minutes before the start of electrical stimulation. Pictures were taken by an 8 megapixel camera (Nikon Coolpix 8700) on a tripod. Constant light condition, manual set of aperture, and exposure time were maintained during the course of the experiment. Pictures of the plantar side of the rat paw were taken 2 minutes after Evans Blue injections, then every 30 seconds during the course of electrical stimulation and for another 5 minutes or longer after the end of electrical stimulation. Matlab image analysis tool (The MathWorks, Inc., MA) was used to determine the dynamic change of color in the development of plasma extravasation. The whole hindpaw was selected as a region of interest. This method was conceptually similar to a dynamic measurement of plasma extravasation by using CCD video camera that has been developed recently [49,50].

### Cutaneous blood flow measurement

Changes in cutaneous blood perfusion were measured in 8 animals to detect local vasodilatation (flare) in response to electrical stimulation of the central stump of the L4 or L5 dorsal roots. The measurements were done using Laser Doppler Imager (PeriScan PIM II, Perimed AB, Sweden). After 10 baseline images of the plantar side of the rat hindpaws, continuous scanning were taken during and after stimulation (20 V, 5 Hz, 0.5 ms pulse duration for 5 minutes) of the central stump of the cut dorsal root. Approximately 20 images were continuously taken after the end of stimulation. It required 2 minutes to acquire one image.

### Data analysis

The stored digital record of unit activity was retrieved and analyzed off-line. The frequency of DRRs was calculated for the periods before (3 min), during (5 min), and after (3 min) the electrical stimulation. Statistical significance was tested using paired t-test.

Matlab software was used in order to measure the intensity of colors on the rat paw. The same region of interest was selected in the set of pictures from each experiment and the change in color intensity in a gray scale was analyzed. The color intensity from Matlab is given as arbitrary unit (AU) for raw data. Normalization was calculated by the following formula: [(color intensity at any time point - color intensity before stimulation) / color intensity before stimulation] × 100%. A negative value represents a darker color, suggesting plasma extravasation. One-way ANOVA followed by Posthoc Fisher LSD Test was used to detect significant differences across time as compared to the baseline.

For blood perfusion, the region of interest was selected which covered the whole paw. An average of perfusion (arbitrary unit, AU) in the selected area of each image frame was used for further calculating percentage change. The first 10 images at baseline were averaged as control for subsequent change during and after stimulation: [(blood perfusion at any time point - average of first 10 blood perfusion images before stimulation) / average of first 10 blood perfusion images before stimulation] × 100%. Repeated measures ANOVA followed by Posthoc Fisher LSD test was used to detect significant differences along the time as compared to the baseline.

All values were presented as means ± SEM. A change was judged significant if p < 0.05.

## Results

### Dorsal root reflexes can be elicited at the central dorsal root filaments by electrical stimulation of a neighboring central stump of the cut dorsal root

**Figure 2:**
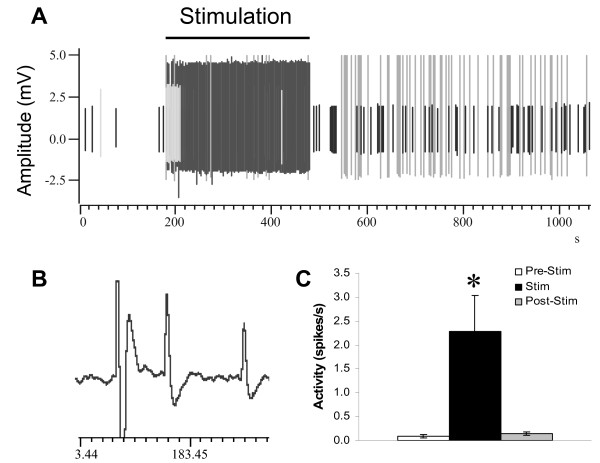
**Dorsal root reflexes from a left L5 filament**. A representative strand shows that DRRs can be recorded from the central stump of the dorsal root (L5) while the L4 central stump is stimulated (A). Each vertical line indicates a DRR. In this strand, about 4 fibers show DRR activity, based on their amplitudes and shapes. The horizontal line indicates the duration of electrical stimulation (20 V, 5 Hz, 0.5 ms for 5 min). During stimulation, an obvious increase of DRRs is demonstrated, which is summarized in (C). An expanded trace is shown in B. *: p < 0.05.

After the left L4 dorsal root was cut, the central stump was placed on the stimulating electrode. To ensure that DRRs can be elicited, a small fascicle of neighboring dorsal root (usually L5) was teased centrally and was placed in a recording electrode (Figure 1A). Multiunit spontaneous antidromic discharges were recorded from all 8 animals that were tested for plasma extravasation. The discharges were irregular and usually at a very low rate but increased during electrical stimulation of L4 (Figure 2A-C). Average mean spontaneous activity was 0.09 ± 0.03 Hz (range: 0-0.22 Hz; n = 8). In most recorded units, additional DRR activity could be evoked by applying a graded mechanical stimulus (brush, pressure, and pinch) to the skin of the foot (data not shown). One cell was found whose receptive field was covering the whole body, as previously reported by others [43,51]. During electrical stimulation (20 V, 5 Hz, 5 ms), a significant increase in DRRs was observed (2.28 ± 0.76 Hz; range: 0.13-5.27 Hz; n = 8, P < 0.05). The activity of antidromic discharges returned to normal 2 min (127 ± 70 s, range from 0 to 585 s) after the termination of electrical stimulation (0.14 ± 0.04 Hz; range: 0-0.36 Hz; n = 8). Four out of eight fibers returned to baseline as soon as the stimulation was terminated; one fiber lasted as long as 10 min.

### Effects of electrical stimulation of the central stump of the cut dorsal root on plasma extravasation on the plantar surface of the ipsilateral and contralateral hindpaws

**Figure 3:**
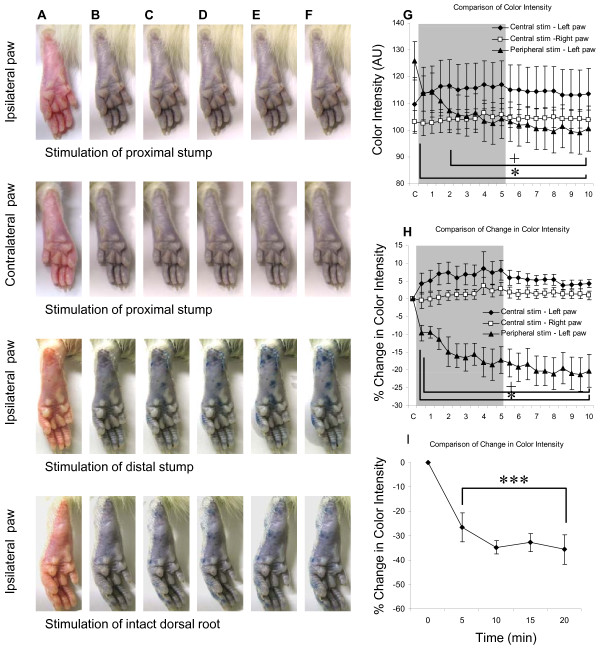
**A series of representative images of the left and right paws while the central stump of cut L4 (1st and 2nd rows) and peripheral stump of cut L5 (3rd row) dorsal root was stimulated**. The blue patches on the paw indicate plasma extravasation due to leakage of Evans Blue (EB). *Note: *A - before EB injection, B - after EB injection, C - 0.5 min, D - 2 min, E - 3.5 min, and F - 5 min after onset of stimulation. A summary shows the changes in color intensity (G) or changes in percentage of color intensity (H) of the ipsilateral (left) or contralateral (right) paws following electrical stimulation of either the central stump of L4 or the peripheral stump of the left L5 dorsal root. When the central stump of the left L4 dorsal root was stimulated, there were no significant differences in the color intensities (G) and percentage changes in color intensity (H) between the left (filled diamond) and right paws (open square). However, significant differences of color intensity and percentage change in color intensity were detected in the left paw when the central stump of L4 (filled diamond) and peripheral stump of L5 dorsal root (filled triangle) were stimulated. A summary of plasma extravasation induced by stimulating intact L5 dorsal root (I) shows significant plasma extravasation (as indicated by the decrease of color intensity, n = 3) 5 min after stimulation. The gray area indicates the duration of stimulation (5 min). *: p < 0.05, ***: p < 0.001, Fisher LSD test, as compared with the color intensity before electrical stimulation. +: p < 0.05, Fisher LSD test, as comparing the peripheral L5 stimulation (left paw) with the central L4 stimulation (left paw). AU: arbitrary unit; C: as a control before stimulation.

When the central stump of the left L4 dorsal root was stimulated, there was no obvious plasma extravasation observed in the ipsilateral (left) paw (Figure 3, 1^st ^row) and contralateral (right) paw (Figure 3, 2^nd ^row). The color intensities before stimulation were 109.57 ± 10.02 in the left paw (Figure 3G) and 103.10 ± 4.25 AU (arbitrary unit) (Figure 3G) in the right paw among 8 animals. During and after central left L4 stimulation, the color intensity for the left paw ranged from 113.72 ± 9.81 to 116.96 ± 8.96 AU (Figure 3G); the color intensity for the right paw ranged from 102.57 ± 4.53 to 106.43 ± 3.46 AU (Figure 3G). Data were analyzed by ANOVA to test differences between sides (ipsilateral and contralateral), and among effects of time (C to 10 min) following central stump stimulation. The results indicated no effect of stimulation side, F (1, 7) = 1.86, p = 0.22; no effect of time, F (20, 140) = 1.33, p = 0.17; and no effect of interaction (Side × Time), F (20, 140) = 1.25, p = 0.23.

By using the color intensity before stimulation to normalize the other data, the percentage change of color intensity of the left paw ranged from 4.31 ± 2.96 to 8.00 ± 2.77% (Figure 3H); the percentage change of color intensity of the right paw ranged from -0.42 ± 2.31 to 3.68 ± 2.42% (Figure 3H). Data were analyzed by ANOVA to test differences between sides of central stump stimulation (side: ipsilateral and contralateral), and among effects of time (time: C to 10 min). The results indicated no effect of stimulation side, F (1, 7) = 5.48, p = 0.052; no effect of time, F (20, 140) = 1.37, p = 0.15; and no effect of interaction (Side × Time), F (20, 140) = 1.23, p = 0.24.

### Effects of electrical stimulation of the peripheral stump of the cut dorsal root on plasma extravasation on the plantar surface of the ipsilateral hindpaw

When the peripheral stump of the left L5 dorsal root was stimulated, there was obvious plasma extravasation observed in the left paw as demonstrated by blue patches (Figure 3A-F, 3^rd ^row). The color intensity of the left paw before stimulation were 109.57 ± 10.02 for central stimulation group (n = 8, Figure 3G) and 125.94 ± 7.10 AU (arbitrary unit) (Figure 3G) for peripheral stump stimulation group (n = 7), respectively. During and after stimulation of peripheral stump of left L5 stimulation, the color intensity for the left paw dropped as low as 99.05 ± 8.06 AU. Data were analyzed by ANOVA to test differences between stimulation site (central vs. peripheral), and among effects of time (time: C to 10 min). The results indicated no effect of stimulation site, F (1, 6) = 0.52, p = 0.5; a significant effect of time, F (20, 120) = 5.29, p < 0.001; and a significant effect of interaction (Site × Time), F (20, 120) = 7.96, p < 0.001. Posthoc Fisher LSD tests indicated significantly lower color intensity in the left paw 2 minutes following stimulation of the peripheral stump of dorsal root (+: p < 0.05) as compared to central stump stimulation (Figure 3G), as well as to before stimulation (*: p < 0.05).

By using the color intensity before stimulation to normalize the other data, the percentage change of color intensity of the left paw dropped to -21.28 ± 4.73% following stimulation of the peripheral stump of dorsal root (Figure 3H). Data was analyzed by ANOVA to test differences between sites of stimulation (central vs. peripheral), and among effects of time (time: C to 10 min). The results indicated significant effect of stimulation site, F (1, 6) = 19.44, p = 0.005; a significant effect of time, F (20, 120) = 4.20, p < 0.001; and a significant effect of interaction (Site × Time), F (20, 120) = 6.16, p < 0.001. Posthoc Fisher LSD tests indicated significantly lower intensity in the left paw following stimulation of the peripheral stump of dorsal root (+: p < 0.05) as comparing to central stump stimulation (Figure 3H), as well as to before stimulation (*: p < 0.05).

### Effects of electrical stimulation of the intact dorsal root on plasma extravasation on the plantar surface of the ipsilateral hindpaw

When the intact left L4 or L5 dorsal root was stimulated, there was plasma extravasation observed in the left paw (n = 3, Figure 3A-F, 4^th ^row). The color intensity change of the left paw was normalized by using the color intensity before stimulation (Figure 3I). The change of color intensity of the left paw were -26.63 ± 5.91 at 5 min, -34.77 ± 2.81 at 10 min, -32.75 ± 3.69 at 15 min, and -35.69 ± 6.16 at 20 min following stimulation, where the negative value indicates an increase in extravasation. Significant changes were found at 5, 10, 15, and 20 min after stimulation. One-way ANOVA showed a significant change after stimulation of the intact dorsal root, F (4, 8) = 31.6, p < 0.001. Posthoc Fisher LSD tests indicated significantly lower intensity in the left paw following stimulation of the intact dorsal root as comparing to before stimulation (***: p < 0.001).

### Effects of electrical stimulation of the central stump of the cut dorsal root on vasodilatation on the plantar surface of bilateral hindpaws

**Figure 4:**
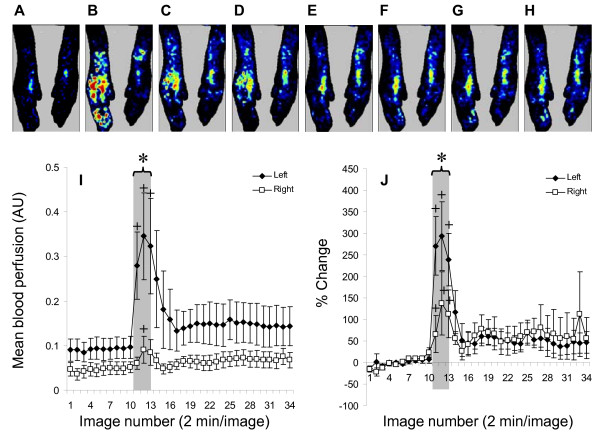
**Effect of stimulation of the central stump of the cut dorsal root on blood perfusion in hindpaws bilaterally**. The upper panel shows laser Doppler images from one rat as control (A), during stimulation (B-D), and 2 (E), 14 (F), 32 (G), and 52 min (H) after the end of stimulation. Blue color indicates lower perfusion, whereas red color indicates higher perfusion. Time response curves are plotted for the raw data (I) and normalized changes in blood perfusion (J) in both the ipsilateral (Left) and contralateral (Right) hindpaws. The gray area indicates duration of stimulation (from image 11 to 13). *Note *+: p < 0.05 as compared to baseline, including all time points under the bracket. *: p < 0.05 as compared to the right side.

When the central stump of left L4 or L5 dorsal root was stimulated, there was obvious vasodilatation in the left paw (Figure 4A-H). The blood perfusion change of the left paw was normalized from raw data (Figure 4I) using the average of 10 baseline images and summarized (ipsilateral n = 8, contralateral n = 5, Figure 4J). Data were analyzed by ANOVA to test differences between hindpaws (ipsilateral vs. contralateral), and among effects of images (image number: 1-34). The results indicated no effect of side of hindpaws, F (1, 3) = 1.66, p = 0.29; a significant effect of images numbers, F (33, 99) = 2.11, p = 0.002; and a significant effect of interaction (Side × Image), F (33, 99) = 2.11, p = 0.002. Posthoc Fisher LSD tests indicated significantly higher blood perfusion in the left hindpaw during stimulation of the central stump of dorsal root as compared to the right hindpaw (*: p < 0.05) (Figure 4I), as well as to before stimulation (+: p < 0.05).

After normalization, an ANOVA to test differences between hindpaws (ipsilateral vs. contralateral), and among effects of images (image number: 1-34) indicated no effect of side of hindpaws, F (1, 3) = 0.16, p = 0.71; a significant effect of images, F (33, 99) = 2.26, p = 0.001; and a significant effect of interaction (Side × Image), F (33, 99) = 2.91, p < 0.001. Posthoc Fisher LSD tests indicated a significant increase of blood perfusion (Figure 4I) in the left hindpaws during stimulation of the central stump of dorsal root as compared to the right hindpaw (*: p < 0.05), as well as a significant percentage increase (Figure 4J) of blood perfusion in both hindpaws as compared to the baseline (+: p < 0.05).

## Discussion

The goal of the present experiment was to minimize confounders introduced by artificial antidromic stimulation of dorsal root or introduction of substances to the periphery by interrupting the communication of the stimulated dorsal root with periphery. In the past, electrical stimulation of one dorsal root elicited DRRs in the neighboring roots and spread along (up to 16 spinal segment in both directions from the stimulated site) and across the spinal cord [52]. This process is believed to operate in all-or-none manner once activated [53]. Since a rat's paw is innervated by L4-L6 originating nerves, we assumed that stimulating the central portion of one cut dorsal root would evoke DRRs to the ipsilateral paw through the remaining two dorsal roots. In fact, in the current experiment electrical stimulation of the central stump of the dorsal root elicited a significant increase in DRR activity in the recorded fibers of the neighboring dorsal roots.

Neuropeptides (particularly, substance P and CGRP) found in peripheral terminals of nociceptive fibers contribute to neurogenic inflammation and are released in response to antidromic stimulation [11,13,40,54]. Therefore, we expected that electrically evoked DRRs in the nerves innervating a rat's paw would produce both plasma extravasation and vasodilation. However, bilateral vasodilation but not plasma extravasation was observed in response to central stump stimulation.

As previously mentioned, SP acting on tachykinin receptors increases microvascular permeability and edema formation [10,13]. CGRP, on the other hand, acting on its receptors produces arteriolar vasodilation [13,15]. Interestingly, C-fibers contain both SP and CGRP, whereas Aδ-fibers predominantly have CGRP in their peripheral terminals [17,55,56]. In addition, antidromic stimulation of the saphenous nerve at C-fiber intensity produces both vasodilation and plasma extravasation, whereas stimulation at Aδ-fiber intensity produces only vasodilation [24,54]. It has been previously shown that 1-2 pulses to lumbosacral dorsal roots are enough to cause a change in cutaneous microcirculation, and 4-16 pulses at 2 Hz evokes vasodilatation lasting for several minutes [20]. Similar results have been shown with spinal cord stimulation [57]. In our study, electrical stimulation of the intact dorsal root or the peripheral stump of the dorsal root produced both vasodilatation and plasma extravasation in the skin. However, electrical stimulation of the central stump with the same parameters did not elicit plasma extravasation on either side, but did produce vasodilatation bilaterally. This finding suggests that the stimulation parameters selected were sufficient to excite both myelinated and unmyelinated fibers in both the distal and central stumps of the dorsal root. However, stimulation of the central stump of the dorsal root triggers more DRRs in myelinated than unmyelinated fibers in the neighboring roots, and leads mostly to CGRP release, and in turn vasodilation.

The differential release of co-localized neurotransmitters from the same terminal depending on the firing rate is another possible explanation of the obtained results. The stimulation frequency needed to induce plasma extravasation is higher than that to produce vasodilation [54]. Electrical stimulation should to some extent mimic peripherally evoked orthodromic action potentials. It is true that DRRs evoked by stimulating the central stump are much weaker than direct stimulation of the distal stump, due to the nature of multisynaptic connectivity inside the spinal cord. It may help to explain the differences in plasma extravasation resulting from stimulation of the central versus peripheral stump. In addition, co-packaged in the same granule, catecholamines and neuropeptides have been shown to be differentially released from adrenal medulla depending on the firing rate through a regulated activity-dependent dilation of the granule fusion pore and size-exclusion mechanism [58,59].

In both of the proposed mechanisms, there should be a higher probability of DRR generation in Aδ-fibers compared to C-fibers in response to central stump orthodromic stimulation. First, there may be a differential effect of GABA on GABA_A _receptors on the central terminals of primary afferents. C-fibers have been shown to have a lower density of GABA_A _receptors compared to both Aδ-fibers and Aβ-fibers [60]. Second, the threshold for generation of DRRs by PADs may be higher in C-fibers compared to Aδ- fibers.

In addition, the proportion of CGRP-containing afferents is much higher compared to SP-containing afferents in the skin. CGRP is present in both myelinated and unmyelinated nociceptive fibers, whereas SP is only found in small diameter unmyelinated fibers. CGRP is also found in larger number of unmyelinated fibers compared to SP [61].

Finally, the role of sympathetic nervous system needs to be addressed, since the stimulation of the central stump may increase sympathetic activity. On one hand, sympathetic activity can decrease neuropeptide release from afferent fibers by its action on prejunctional α_2_-adrenoreceptors [13], and counteract dorsal reflex-mediated neurogenic inflammation [62]. On the other hand, sympathetic presence is important for the development of DRR-mediated neurogenic inflammation through the actions of neuropeptide Y (NPY) and norepinephrine on NPY Y_2 _and alpha_1 _receptors, respectively [63,64].

In this study, the contribution of the sympathetic nervous system during central stump stimulation was challenged by two experiments: blood perfusion change in stimulation of the central stump of the cut dorsal root (Figure 4) and plasma extravasation in stimulation of intact dorsal root (Figure 3I). Stimulation of the central stump produced a significant bilateral increase in blood perfusion suggesting that DRRs in primary afferents surpass sympathetic vasoconstriction, if present. Stimulation of intact dorsal root on the other hand (action potentials can travel orthodromically and antidromically) produced plasma extravasation in the ipsilateral hindpaw, suggesting that even if sympathetic system is activated by orthodromic input, its subsequent effects are not strong enough to counteract plasma extravasation induced by the antidromic spikes that reached the periphery.

## Conclusion

In summary, incoming stimulation at an intensity that activates all types of nociceptive fibers produces DRRs in the intact neighboring roots as well as bilateral vasodilation of the innervated area but not plasma extravasation. Neurogenic inflammation is a complex process that requires the co-release of multiple substances. It seems that noxious stimulation alone is not capable of eliciting all signs of neurogenic inflammation. Therefore, successful treatment of neurogenic inflammation will not only require that the neural input to the spinal cord be addressed, but also that co-factors in both spinal cord and the periphery that allow neural input to convert to neuropeptide co-release be addressed. In addition, acutely elicited DRRs are not able to elicit the complete picture of neurogenic inflammation; future studies are necessary to establish the contributions and nature of DRRs in chronic pain states such as arthritis or migraine.

## Competing interests

The authors declare that they have no competing interests.

## Authors' contributions

This study is based on the original idea of OVL and YBP. OVL performed data collection, data analysis, the statistical analysis and wrote the manuscript. YBP made contributions to conception and design and analysis and interpretation of data. All authors have read and approved the final manuscript.
